# Exclusion of prognostic coronary disease in LV dysfunction using late gadolinium enhancement CMR

**DOI:** 10.1186/1532-429X-17-S1-P187

**Published:** 2015-02-03

**Authors:** Alexandra Thompson, Jenifer Crilley, Jerry J Murphy, Douglas Wilson, Pali Hungin, Ahmet Fuat

**Affiliations:** Cardiology, Darlington Memorial Hospital, Tyne and Wear, UK; School of Medicine, Pharmacy and Health, Durham University, Durham, UK

## Background

In new diagnoses of left ventricular (LV) systolic dysfunction it is important to exclude coronary artery disease (CAD) as the cause. Cardiac magnetic resonance imaging using late gadolinium enhancement (LGE CMR) highlights fibrosis from ischaemia or infarcts in a subendocardial distribution. Many consider that the absence of LGE indicates an absence of significant CAD. The evidence for this is debatable, with variable sensitivities depending upon the definition of CAD, the patient population, and the use of proximal coronary artery imaging (MRCA). We strived to ascertain the utility of LGE CMR to exclude prognostic CAD.

## Methods

We retrospectively reviewed a cohort of patients attending a district general hospital who underwent both X-ray angiography and LGE CMR since 2006. Records of those with European criteria for LV systolic dysfunction (LVEF <50% or LVEDVI ≥97mL/m^2^) on CMR or transthoracic echo were analysed. The presence and extent of subendocardial LGE was recorded with the 17 segment model. The degree of coronary stenosis at X-ray angiography was assessed visually and significant disease defined as stenosis of the LMS ≥50%, or proximal LAD ≥75%, or ≥70% in two main coronary vessels.

## Results

A total of 116 patients were included. Mean age was 64 years and 78% were male. Mean LVEF was 40%. The prevalence of prognostic CAD was high at 47%. Subendocardial LGE detected prognostic CAD with a sensitivity of 100% (95% CI, 94 to 100%) with no false negative results.

## Conclusions

LGE CMR reliably excludes prognostic CAD in patients with LV systolic dysfunction.

## Funding

N/A.

**Table 1:** Diagnostic parameters of LGE CMR to predict prognostic CAD

	Performance of LGE CMR (95% Confidence Interval)
Prevalence of prognostic CAD	47% (38 to 57%)
Sensitivity	100% (94 to 100%)
Specificity	44% (32 to 58%)
Positive Predictive Value	62% (51 to 72%)
Negative Predictive Value	100% (87 to 100%)
False Omission Rate	0% (0 to 13%)

LGE CMR, Cardiac magnetic resonance with late gadolinium enhancement sequences; CAD, Coronary artery disease. Results confirmed by exact methods.

**Table 2:** Diagnostic performance of LGE CMR to predict prognostic CAD

CMR LGE	X-ray angiogram		Total
	Prognostic CAD present	Prognostic CAD absent	
Subendocardial LGE present	55 (TP)	34 (FP)	89
Subendocardial LGE absent	0 (FN)	27 (TN)	27
Total	55	61	116

CAD, Coronary artery disease; CMR, Cardiac magnetic resonance; LGE, Late gadolinium enhancement; TP, True positive; FP, False positive; FN, False negative; TN, True negative.

